# The effects of sex and hormonal status on restraint-stress-induced working memory impairment

**DOI:** 10.1186/1744-9081-2-8

**Published:** 2006-03-07

**Authors:** Rebecca M Shansky, Katya Rubinow, Avis Brennan, Amy FT Arnsten

**Affiliations:** 1 Yale University School of Medicine, Dept of Neurobiology, 333 Cedar St, New Haven, CT 06510, USA

## Abstract

**Background:**

Restraint stress has been shown to elicit numerous effects on hippocampal function and neuronal morphology, as well as to induce dendritic remodeling in the prefrontal cortex (PFC). However, the effects of acute restraint stress on PFC cognitive function have not been investigated, despite substantial evidence that the PFC malfunctions in many stress-related disorders.

**Methods:**

The present study examined the effects of restraint stress on PFC function in both male rats and cycling female rats in either the proestrus (high estrogen) or estrus (low estrogen) phase of the estrus cycle. Animals were restrained for 60 or 120 minutes and then tested on spatial delayed alternation, a PFC-mediated task. Performance after stress was compared to performance on a different day under no-stress conditions, and analyzed using analysis of variance (ANOVA).

**Results:**

Sixty minutes of restraint impaired only females in proestrus, while 120 minutes of restraint produced significant impairments in all animals. Increases in task completion times did not affect performance.

**Conclusion:**

These results demonstrate an interaction between hormonal status and cognitive response to stress in female rats, with high estrogen levels being associated with amplified sensitivity to stress. This effect has been previously observed after administration of a pharmacological stressor (the benzodiazepine inverse agonist FG7142), and results from both studies may be relevant to the increased prevalence of stress-related disorders, such as major depressive disorder, in cycling women. Overall, the results show that restraint stress has important effects on the cognitive functions of the PFC, and that hormonal influences in the PFC are an important area for future research.

## Background

Exposure to both single and multiple restraint sessions has been a classic model of stress for over three decades. Restraint stress induces a number of changes in many brain regions, including suppression of long term potentiation and reduced neurogenesis in the dentate gyrus [1,2], dendritic remodeling in hippocampus (reviewed in [3]) and prefrontal cortex (PFC) [4,5] as well as hormonal, biochemical and molecular changes throughout the brain (reviewed in [6]). Restraint stress can also alter an animal's performance on several cognitive tasks, including passive avoidance [7], fear conditioning [8] and the Radial Arm Maze [9]. To date however, restraint stress has not been used to challenge working memory ability. Spatial delayed-alternation in a T maze is a classic test of PFC function in that it demands updating of information, inhibition of a tendency to return to a previously rewarded location, and concentration during the delay period [10,11]. Previous studies have shown that administration of a pharmacological stressor--the anxiogenic, partial inverse benzodiazepine agonist FG7142 – impairs delayed alternation performance [12-14].

This lab has also recently demonstrated that females in proestrus are more sensitive than males and females in estrus to the PFC-impairing effects of pharmacological stress (benzodiazepine inverse agonist FG7142) [15]. These results suggest that the high circulating levels of estrogen during this phase can influence the prefrontal cortical cognitive response to stress. However, there have been no studies examining sex differences on prefrontal cortical function using classical stressors such as restraint stress. In contrast, there have been several important studies showing that sex differences have a large influence on the response to restraint stress using behavioral paradigms that do not depend on the prefrontal cortex. For example, Wood and Shors [16] showed that acute restraint stress impaired classical eyeblink conditioning in intact females, but not ovariectomized females or males. In intact females, the most severe impairment occurred during proestrus, when estrogen levels were highest. Eyeblink conditioning has been shown to involve cerebellar circuits [17], and possible hippocampal involvement as well [18]. In contrast, very different results are observed when animals are challenged with spatial tasks dependent on the hippocampus. Female rats are reported to be unaffected or even enhanced by exposure to stress regimens that normally produce impairments in males on hippocampally-mediated tasks like the Y-maze [19] and radial arm maze [20]. These results indicate that the influence of ovarian hormones on the stress response depends on the nature of the stress (acute or chronic), and the brain circuits engaged during cognitive assessment.

The current study tested the hypothesis that acute restraint stress would produce working memory deficits in male and female rats in a pattern similar to those seen after pharmacological stress. Indeed, we report that females with high levels of circulating estrogen were more sensitive to stress-induced impairments than males or females with low estrogen levels.

## Materials and methods

### Subjects

Male and female Sprague-Dawley rats approximately 240–260 g in weight and 2 months in age, (Taconic, NY) were single-housed in a 12 hr light/dark cycle with all testing conducted during the light phase. The animals were fed Purina rat chow (15 g/rat/day) immediately following behavioral testing and water was available *ad libitum*).

### Estrus phase monitoring

After testing each day (approximately 12 pm), females were vaginally lavaged, and the cells were spread on a microscope slide. Cells were stained with Cresyl Violet, covered, and examined under a light microscope in order to determine estrus cycle phase. Proestrus cells are large, have small nuclei, and often are found in organized clumps. Estrus cells are cornified. Metaestrus cells are darkly stained and cornified, and diestrus cells are round and nucleated. Only animals that were cycling normally (4–5 day cycle) were restrained. Animals were restrained only during proestrus or estrus, so as to reduce the number of restraint sessions for each animal, as well as to replicate earlier findings from this lab and others that demonstrate divergent effects of stress in these two phases [15,21]. Only animals that were cycling regularly were included in the study.

### Cognitive testing

Delayed alternation training and testing were performed in a T-maze (laquered plywood, w: 90 cm × l: 65 cm × h: 8 cm). Rats were habituated to the T-maze until they were readily eating chocolate chips from the experimenter's hand. PFC cognitive function was measured by the spatial working memory task delayed alternation. This task requires working memory, behavioral inhibition and sustained attention, and has been shown to be impaired in animals with ventromedial PFC lesions (specifically, infra- and pre-limbic areas) [10], as well as in rats administered the pharmacological stressor FG7142 [13]. Following habituation, rats were trained on the delayed alternation task. A rat was placed in the start box of the T-maze and the gate was opened, allowing the rat to run to the choice point in the maze. On the first trial each day, animals were rewarded (fed a chocolate chip from the tester's hand) for entering either arm. The rat was then picked up and returned to the start box of the maze for the intertrial delay. On all subsequent trials the rat was rewarded only if it entered the maze arm that was not chosen on the immediately preceding trial. If the correct choice was made, the rat was given a reward and returned to the start box for the intertrial delay. Following an incorrect choice, the rat was immediately returned to the start box for the intertrial delay without reward. During the inter-trial delay, the maze was wiped with 75% ethanol to remove any olfactory cues. Each test session consisted of 10 trials. Rats were scored for accuracy of response and response time. Response time was measured from the time the start gate was lifted until the animal made its choice. Rats were tested once daily, at the same time of day, 5 times per week, for the duration of the study (approximately 6 months). Please note that impairment on this task is reflected by performance of ~5 correct, which represents chance level of responding. A score lower than this indicates perseverance towards one arm of the maze.

The intertrial delay was initially ~2 s, the minimal time needed to clean the maze. Delays were raised by 5 s increments as needed in order to stabilize each rat's performance at approximately 7 out of 10 correct. This score was used as a baseline in order to ensure against ceiling effects, and so that either impairment or improvement may be observed after stress exposure. After 60 days of testing, animals did not differ between groups in level of delay (range 5–20 s). Animals were tested daily until a rat scored between 6 and 8 correct for 2 consutive days; however, 2 consutive days with a score of 6 was not acceptable. On the following day, animals were restrained (or received the "cage" control treatment) if their estrus phase was predicted to be either proestrus or estrus. Since estrus phase was confirmed after testing (to avoid interference with performance), if an animal proved not to be in proestrus or estrus after having been restrained, that data point was discarded.

### Restraint

**Figure 1:**
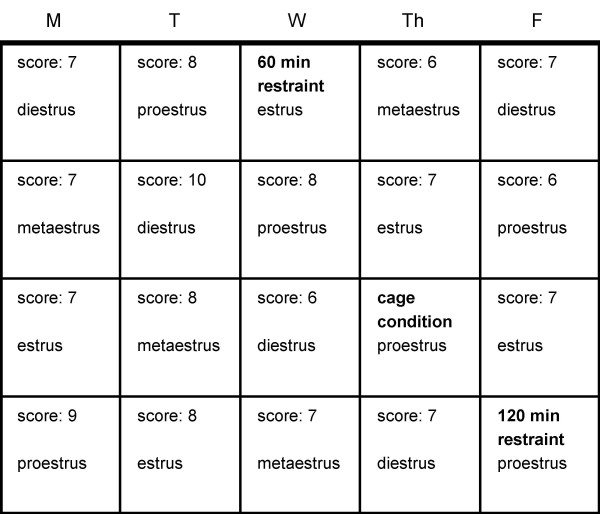
Representative Training Schedule. Rats were tested daily, Monday through Friday. In order to be eligible for stress testing, three criteria must be met: 1) The animal must have achieved a score of 6, 7 or 8 (out of 10) for at least two consutive days; 2) at least one week must have passed since the last stress exposure; 3) females must be cycling regularly on a 4–5 day cycle, and it must be anticipated that they will be in either proestrus or estrus on stress day. In this figure, the rat is ready for testing on the first Wednesday because her two previous scores are 7 and 8, and she will be in estrus. In the sond week, she does not receive stress treatment because her scores do not qualify her on a day when she will be in estrus or proestrus. In the third week, her scores on Tuesday and Wednesday qualify her for treatment on Thursday, and she will be in proestrus, so she receives treatment. In week four, her scores and cycle qualify her for treatment on Friday.

Animals were restrained in plastic restraint devices (Harvard Apparatus) for 60 min, 120 min or were left in their home cage in the testing room for 1 hour prior to testing. Animals were tested on the T-maze task immediately after release from restraint. It should be noted that this is different from many studies, which often measure the effects of stress 24 hr after stress exposure. However, the decision to conduct the current study as such was made in order to be consistent with all previous work in this lab. To avoid potential habituation to restraint, at least one week passed between restraint sessions for each animal, and the order in which each animal received each treatment (including the cage condition) was randomized. After a stress exposure, animals were tested daily on the T-maze task for at least one week, and animals must reach stable baseline criteria again before being considered eligible for the next stress exposure. See figure 1 for a representative testing schedule.

### Analysis

Data were analyzed using analysis of variance (ANOVA. Planned comparisons were performed with a test of effects using Systat software.

## Results

### Effects of restraint stress on accuracy of delayed alternation performance

**Figure 2:**
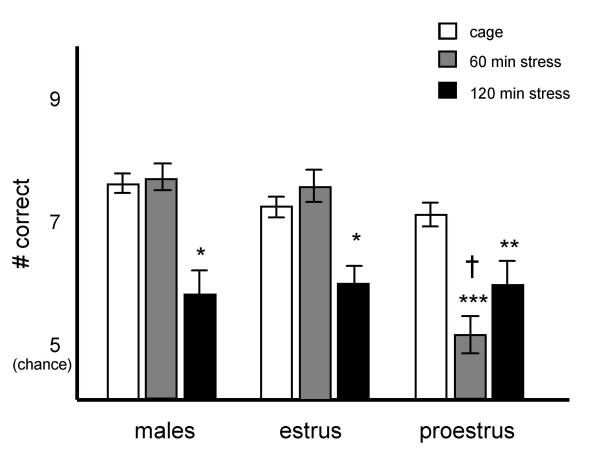
**Females in proestrus are more sensitive to the PFC-impairing effects of stress than males or females in estrus**. Animals were restrained for 0, 60 or 120 min prior to testing on the delayed alternation T-maze task. Results are represented as mean +/- SEM number correct. Mean scores after 0, 60 and 120 min restraint were 7.4 +/- .22, 7.6 +/- .3 and 5.7 +/- .51 for males; 7.1 +/- .18, 7.4 +/- .4 and 5.9 +/- .51 for females in estrus; and 6.9 +/- .2, 5 +/- .35 and 5.9 +/- .5 for females in proestrus. * = significantly different from self in control conditions, p < .05, ** = p < .005, *** = p < .0005, † = significantly different from self during estrus, p < .02.

The effects of restraint stress on performance of the spatial delayed alternation task were examined in males (n = 7), and in cycling females in either estrus (n = 5) or proestrus (n = 5). An ANOVA with repeated measures analyzed the influence of sexual status (between subjects factor: males, females in proestrus, females in estrus) on the effects of restraint stress (within subjects factor: 0, 60 or 120 min or restraint). Data are shown in Figure 2. This analysis revealed a significant between subjects effect of sexual status (F [2.14] = 6.64, p < .01); a significant within subjects effect of restraint (F[2,14] = 7.31, p < .003); and a significant sexual status × restraint interaction (F[4,28] = 3.19, p < .03). Tests of effects revealed that there were significant sex differences in performance only after 60 min of restraint (F[2,14] = 14.0, p < .0005). As can be seen in Figure 2, females in proestrus were impaired by 60 min restraint while males and females in estrus were not. All animals were impaired by 120 min restraint. There were no significant sex or estrus effects during control conditions or after 120 min restraint (p > 0.1).

### Effects of stress on response time

**Figure 3:**
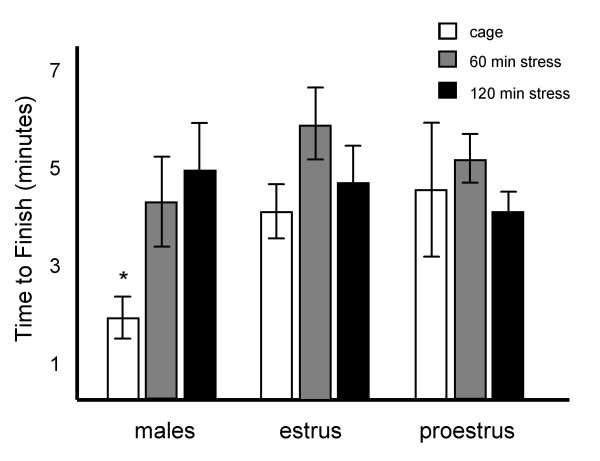
**Group differences in time were observed, but they did not correlate with differences in performance**. Task completion times were recorded for all groups. Results are represented as mean +/- SEM minutes to finish. Mean task completion times (in minutes) after 0, 60 and 120 min were 1.8 +/- .4, 4.5 +/- 1.1 and 5 +/- 1 for males; 4.3 +/- .5, 5.8 +/- .7 and 4.8 +/- .64 for females in estrus; and 4.7 +/- 1.6, 5.3 +/- .6 and 3.9 +/- .4 for females in proestrus. * = significantly different from all other groups.

Mean times-to-finish after 0, 60 or 120 min restraint stress are represented for the males, females in prostrus and females in estrus in figure 3.

A repeated-measures ANOVA revealed a significant within subjects effect of restraint on response time (F[2,26] = 6.8, p < .005), consistent with increased freezing responses following stress exposure in rodents. There was no significant between subjects effect of sexual status on response time (F[2,13] = .017, p = .0.84), and a small but significant sex by restraint interaction (F[4,26] = 3.14, p = 0.03). Tests of effects showed that this interaction arose from response time differences during the control condition (no restraint) in which males tended to be faster than the females (F[2,13] = 3.19, p = 0.075). In contrast, there were no differences in response time following 60 min restraint (F[2,13] = .48, p = 0.63) or 120 min restraint (F[2,13] = .77, p = 0.48). Importantly, there was no correlation between time-to-finish and performance for any group (males r = -0.38, p > .05, estrus r = 0.09, p > .05, proestrus r = -0.25, p > .05), indicating that slower response time did not predict worse performance.

## Discussion

These data demonstrate that exposure to restraint stress can impair spatial delayed alternation performance. These results extend previous work showing that an anxiogenic benzodiazepine inverse agonist, FG7142, can similarly impair performance of this task. FG7142 has no effect on a control task, spatial discrimination, with similar motor and motivational demands, but not requiring working memory or PFC cognitive function [12]. Thus, stress-induced deficits in delayed alternation performance appear to reflect impairments in working memory operations dependent on the PFC. These findings complement the growing literature demonstrating that restraint stress can alter performance of behaviors dependent on amygdala and hippocampus [8,9].

A more noteworthy finding of this study was the identification of differences in sensitivity to restraint stress across the estrus cycle, where females in proestrus, but not estrus, were impaired by 60 min restraint. These data support previous work from this lab showing that a benzodiazepine inverse agonist, FG7142, was more effective at eliciting PFC dysfunction during proestrus than during estrus [15]. As proestrus is characterized by high levels of estrogen, this work provides further evidence that estrogen can act to promote sensitivity to the PFC-impairing effects of stress. As expected, all animals demonstrated significant impairment with more severe stress, 120 min of restraint. This again mimics results from previous work, which shows that larger doses of FG7142 can cause impairment regardless of sex or estrus phase [14,15]. In contrast, Figueiredo et al [22] reported that animals in proestrus show a muted acute stress response (as measured by c-Fos expression) compared to that of males or females in estrus or diestrus. However, this study saw its most robust c-Fos induction in the cingulate and motor regions of the frontal cortex, with virtually no changes in the pre- or infralimbic regions in any group. The delayed alternation task is mediated by these latter two regions [11]; thus stress-induced PFC dysfunction is likely manifest through different mechanisms or pathways than those associated with stress-induced c-Fos expression.

The increased sensitivity to stress in proestrus does not appear to be an artifact of group differences in time to complete the task. Specifically, it might be argued that an animal that takes longer to make each choice might be at a disadvantage in remembering which arm it had previously chosen, and perform more poorly than quicker animals. Estrogen and stress have been shown to affect locomotor behavior as measured by the open field test [23], which could potentially confound the results of our study. However, there were no significant differences in task completion times between groups whose performance differed with stress, nor were there baseline differences in task completion times between animals in estrus and proestrus, suggesting a dissociation between these factors. Thus, locomotor activity likely did not affect cognitive performance in this paradigm. These results were also not likely due to differences in spatial ability, as acute pharmacological stress has been shown to have no effect on a T-maze spatial discrimination task [14]. Moreover, it has been demonstrated that female rats' spatial ability can in fact be enhanced by acute restraint stress [19]; thus stress effects on spatial ability are likely not contributing to the pronounced cognitive deficits observed here.

One factor that warrants consideration is the potential effect of progesterone on the results obtained in this study. In addition to estrogen, progesterone levels fluctuate with the estrus cycle, with high levels during estrus, and rising levels during proestrus. However, ovariectomized animals with only estrogen replacement have shown a sensitivity to stress comparable to animals in proestrus as currently reported [15], suggesting that the primary effect is due to changes in estrogen levels. That said, the potential role of progesterone in modulating stress effects should be the subject of future experiments.

The present results may also be due in part to estrogen-corticosterone interactions. Sex differences have been found with respect to basal levels as well as stress-induced release of corticosterone, with female rats releasing more corticosterone than males after 60 min of restraint, and having greater basal levels during proestrus than diestrus [24]. Corticosterone is released into the PFC during stress, but its contribution to stress-induced PFC impairment has yet to be thoroughly described. Recent work suggests that corticosterone can, indeed, disrupt working memory [25], but the mechanisms by which this occurs are not known. Future experiments will address this issue.

The present study provides further evidence that female rats in proestrus are more sensitive to the PFC-impairing effects of acute stress. These findings hold clinical relevance in that stress-related mental illnesses such as Major Depressive Disorder (MDD), often characterized by abnormalities in PFC morphology and function [26-28], are more prevalent in women than in men [29]. Moreover, this gender discrepancy appears at puberty, maintains through childbearing years, and then declines after menopause [30], suggesting that circulating estrogen might make women more susceptible to stress-induced dysfunction. Much work is needed before the exact nature of estrogen's role in the stress response will be fully understood, much less yield clinical applications. That said, stress-related disorders continue to be a major public health concern for women, and basic research plays an important role in understanding the biological mechanisms underlying these disorders.

## List of abbreviations

All abbreviations are defined within the text.

## Competing interests

The author(s) declare that they have no competing interests.

## Authors' contributions

AA and RS designed the study, performed the statistical analysis and drafted and revised the manuscript. RS, KR and AB carried out the behavioral testing and estrus monitoring. All authors read and approved the final manuscript.
